# Gradient-Field-Based Force-Driven Control of a Mudskipper-Inspired Magnetic Microrobot for Intestinal Applications

**DOI:** 10.3390/mi17040476

**Published:** 2026-04-15

**Authors:** Yijie Du, Huiting Xie, Wenqi Zhang, Yuting Mao, Gongxin Li

**Affiliations:** The Key Laboratory of Advanced Process Control for Light Industry (Ministry of Education), Institute of Automation, Jiangnan University, Wuxi 214122, China

**Keywords:** magnetic microrobot, gradient magnetic field, force-driven control, confined-space navigation, mucosal elevation, intestinal applications

## Abstract

Magnetically driven microrobots operating in intestinal environments face two major challenges: difficulty in traversing low-height confined spaces and limited local visibility caused by mucosal obstruction. To address these issues, this study proposes a gradient-field-based force-driven control method for a mudskipper-inspired magnetic microrobot. By establishing the mapping among coil current, magnetic field, and magnetic force at the robot working point, and by solving the control input through singular value decomposition and linear programming, effective magnetic-force output along a desired direction was achieved. On this basis, two representative force-driven motions were designed. The first was a translational mode based on pulsed magnetic-force actuation for stable navigation in low-height confined spaces. The second was a lifting mode based on continuous loading and gradual adjustment of the magnetic-force upper bound to locally lift a flexible “mucosa-like” membrane, thereby simulating intestinal mucosal elevation and local visual field expansion. Experimental results showed that the robot could stably pass through narrow tunnels and effectively lift an overlying flexible membrane under vertical magnetic-force actuation. The proposed method extends both the locomotion capability and the local interaction capability of the mudskipper-inspired magnetic microrobot, and demonstrates a feasible proof-of-concept approach for confined-space navigation and localized manipulation in intestinal applications.

## 1. Introduction

With the rapid development of minimally invasive medicine and intelligent medical devices, interventional and surgical robots are evolving toward miniaturization, flexibility, and high precision. Compared with conventional rigid surgical instruments, such robots can enter natural body cavities or narrow anatomical spaces and perform inspection, localization, drug delivery, biopsy, and local treatment in the gastrointestinal tract, vasculature, respiratory tract, and other complex regions. Therefore, they show significant potential for improving operational safety, reducing tissue trauma, and enhancing the accessibility of diagnosis and treatment [1,2,3]. In recent years, a variety of actuation strategies have been proposed for capsule endoscopes, flexible endoscopes, continuum robots, and micro/nanorobots, including optical actuation [4,5], acoustic actuation [6], electrical actuation [7], chemical actuation [8,9], biohybrid actuation [10,11], and magnetic actuation [12,13,14]. Among these methods, optical actuation offers high spatiotemporal resolution and remote controllability, but its penetration capability in deep tissue is limited [15,16]. Ultrasound-based actuation is non-contact, non-invasive, and biocompatible, making it attractive for in vivo applications, although its manipulation accuracy still requires further improvement [17,18]. Electrical actuation has advantages in microscale assembly and manipulation, but its clinical translation is constrained by electrode dependence and limited adaptability to biological environments [19,20]. Among the available strategies, magnetic actuation has emerged as one of the most representative approaches for miniature medical robots because it enables wireless remote control, provides strong penetration through biological tissues, adapts well to non-transparent media, and offers good biosafety [21].

Existing magnetically actuated miniature medical robots can be broadly categorized into capsule robots [22,23], continuum robots [24], and microrobots [25,26,27], and have been investigated for a wide range of clinical applications in the cardiovascular, respiratory, gastrointestinal, and urinary systems. Representative tasks include medical inspection [28], targeted drug delivery [29,30], lesion ablation [31], biopsy [32], and stent delivery [33]. Nevertheless, several key challenges remain. These include the limited workspace of external magnetic actuation systems, insufficient actuation efficiency and cross-scale locomotion capability, the need for improved biocompatibility and biodegradability of robotic materials, difficulties in system miniaturization and multifunctional integration, and limited stability and robustness of closed-loop control in complex in vivo environments [34,35,36,37]. Therefore, systematic studies on magnetic actuation mechanisms, structural design, imaging feedback, and closed-loop control are essential for advancing magnetic medical robots from laboratory demonstrations toward practical interventional applications.

To address these issues, this study focuses on a mudskipper-inspired magnetic microrobot for intestinal applications. This robot was selected because its design is inspired by the anatomical and kinematic characteristics of the mudskipper, which is known for its strong mobility over complex terrains such as tidal flats. The robot employs a pair of semi-circular magnetic feet inspired by the mudskipper’s pelvic fins, and its locomotion resembles the crawling mechanism of the mudskipper. Under a uniform magnetic field, this structure exhibits favorable crawling capability and strong terrain adaptability, and previous work has verified its stable locomotion on complex surfaces. However, when considered for intestinally relevant scenarios, two critical limitations remain. First, the intestine contains many low-height and confined spaces in which the conventional crawling gait cannot be fully deployed, thereby restricting the robot’s traversability. Second, during colonoscopic procedures, mucosal folds may obstruct the surgical field of view, which creates a need for a robot capable of elevating the mucosa to provide local visual field expansion. To this end, a magnetic-force-driven control method based on gradient magnetic fields is proposed. By establishing the mapping among coil current, magnetic field, and magnetic force at the robot working point, and by solving the control input through singular value decomposition and linear programming, effective magnetic-force output along a desired direction is achieved. On this basis, two representative force-driven motions are developed: translational motion for stable navigation in low-height confined spaces and lifting motion for local elevation of a flexible membrane. Finally, narrow-tunnel navigation experiments and membrane-lifting experiments are conducted as proof-of-concept validations of the proposed method, demonstrating a feasible approach for confined-space navigation and local visual field expansion of magnetic microrobots in intestinal applications.

## 2. Materials and Methods

### 2.1. Fabrication of the Microrobot

**Figure 1:**
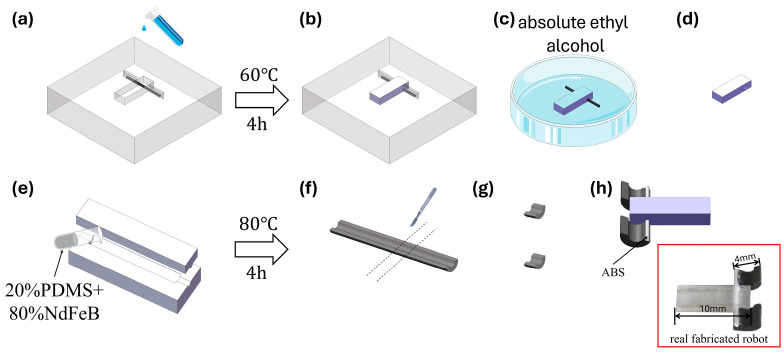
Fabrication and assembly process of the mudskipper-inspired magnetic microrobot. (**a**) Placement of a tungsten rod in the mold and casting of PDMS precursor. (**b**) Thermal curing at 60 °C for 4 h. (**c**) Immersion of the cured sample in anhydrous ethanol to reduce rod–PDMS adhesion. (**d**) Removal of the tungsten rod. (**e**) Casting of the NdFeB/PDMS mixture (8:2 by mass) into a semi-circular mold. (**f**) Thermal curing at 80 °C for 4 h. (**g**) Cutting into 3 mm strips to form the magnetic feet. (**h**) Final assembly by inserting an ABS rod through the channel and fixing the feet to the rod; the inset shows a photograph of the fabricated robot.

The microrobot used in this study is a mudskipper-inspired magnetic microrobot composed of a flexible body, a connecting component, and two symmetric magnetic feet. The body provides structural support and internal space for assembly, while the magnetic feet generate magnetic torque and magnetic force under an external magnetic field and therefore serve as the key functional components for magnetic-force-driven locomotion. The fabrication and assembly process of the robot is illustrated in Figure 1 and mainly consists of three steps: body fabrication, magnetic-foot fabrication, and final assembly.

The robot body was fabricated from polydimethylsiloxane (PDMS, DOWSIL 184, Dow Chemical, Midland, MI, USA). During fabrication, a polytetrafluoroethylene (PTFE) mold machined by a CNC machine was first prepared, and a tungsten rod with a diameter of 0.8 mm was placed inside the mold to reserve a circular channel within the body. The prepared PDMS was then cast into the mold and thermally cured at 60 °C for 4 h to obtain the flexible body. The PTFE mold provided good demolding performance, which facilitated intact removal of the cured body, while the tungsten rod was used to form the internal channel required for the subsequent connecting component. After curing, the sample was immersed in anhydrous ethanol to reduce the adhesion between the tungsten rod and the PDMS, allowing the rod to be removed without damaging the body and thus leaving a through-channel inside the robot.

The magnetic feet were fabricated from a composite of neodymium–iron–boron (NdFeB) magnetic powder and PDMS. The NdFeB powder had an average particle size of 5 μm (MQP-15-7-20065-089, Tianjin Magnequench Co., Ltd., Tianjin, China). To balance the load-bearing capability and post-curing toughness, the NdFeB powder and PDMS were mixed at a mass ratio of 8:2. A relatively high magnetic-powder fraction helped increase the magnetization density of the feet, enabling sufficient magnetic driving force and magnetic torque under external magnetic fields, whereas PDMS preserved the toughness of the cured magnetic feet and provided a certain degree of compliance. The composite mixture was poured into a pre-fabricated PTFE mold and thermally cured at 80 °C for 4 h. After demolding, the cured material was cut with a scalpel into magnetic feet with a width of 3 mm, which served as the locomotion units on the left and right sides of the robot. The feet were then magnetized along the chord direction under a magnetic field of 2 T.

After the body and magnetic feet had been fabricated separately, an acrylonitrile butadiene styrene (ABS) rod with a diameter of 0.6 mm was used as the connecting component for final assembly. During assembly, the ABS rod was first inserted through the reserved channel in the robot body, and the two symmetric semi-circular magnetic feet were then fixed to the two ends of the rod, forming the complete robot structure. In this configuration, the ABS rod connected the body and the two magnetic feet and maintained a stable relative position between them, thereby providing mechanical support for force transmission and posture variation under magnetic actuation.

The locomotion performance of the robot depends not only on the external magnetic field conditions but also on the dimensions of the magnetic feet, the NdFeB content, and the assembly symmetry of the two sides. Therefore, during fabrication and assembly, particular attention was paid to ensuring consistent foot mass, symmetric installation, and accurate alignment of the internal channel and connecting component, so as to reduce the influence of structural asymmetry on locomotion stability. The assembled robot combines a compliant body with magnetic responsiveness and provides the experimental platform for the subsequent translation, lifting, and mode-switching experiments.

### 2.2. Experimental Instruments and Magnetic Control System

**Figure 2:**
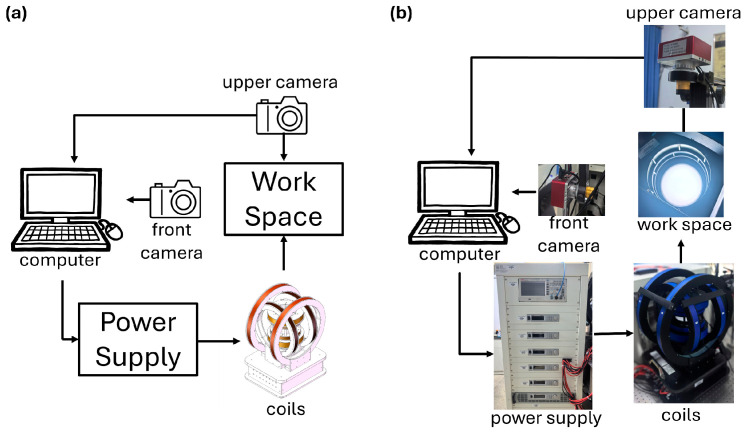
Control system layouts. (**a**) Schematic layout of the control system. (**b**) Physical layout of the control system.

To realize magnetic actuation and experimental observation of the robot, an experimental platform was established, consisting of a magnetic field generation system, a vision module, a power-driving module, and a host control unit. By adjusting the output currents, the platform can generate controllable magnetic fields and gradient magnetic fields within the workspace, while real-time visual localization is used to monitor the robot motion. This platform provides the hardware basis for the subsequent force-driven motion control experiments. The overall configuration of the platform is shown in Figure 2a.

The external magnetic field was generated by a tri-axial Helmholtz coil system together with a pair of Maxwell coils. The tri-axial Helmholtz coils were used to provide magnetic field components in different directions within the workspace, mainly for magnetic moment alignment, posture adjustment, and horizontal force-driven control. In addition, they could also generate gradient magnetic fields to support translational motion of the robot. The Maxwell coils were used to generate a vertical gradient magnetic field to provide the normal magnetic force required for lifting and membrane-elevation tasks. By adjusting the currents applied to each coil set, the system could generate controllable magnetic field intensity and field gradients at arbitrary points within the workspace, thereby supporting the force-driven motion control investigated in this study.

The power-driving module provided stable, continuous, adjustable, and reversible current output to the coils. As shown in Figure 2b, the coil connections were configured according to their functions. The two coils in the X- and Y-direction Helmholtz pairs were powered independently to enable flexible regulation of the magnetic field and field gradients in the horizontal plane. The Z-direction Helmholtz pair was connected in series with the same current direction to generate a uniform vertical magnetic field, whereas the Z-direction Maxwell pair was connected in series with opposite current directions to produce a vertical gradient magnetic field. Through this configuration, the system provided six independent control degrees of freedom, enabling three-dimensional regulation of both magnetic field intensity and magnetic field gradient to satisfy the requirements of different actuation tasks.

To enable real-time observation of the robot motion, an adjustable backlight was placed beneath the central workspace of the coil system to provide transmitted illumination. Two cameras (WST-4K60FPS, Shenzhen Weishite Trading Co., Ltd., Shenzhen, China) were mounted above and beside the workspace, respectively. The top-view camera was mainly used for planar observation and robot localization, while the side-view camera was used to record posture variation and side-view deformation during lifting and membrane-elevation processes. This dual-view configuration allowed more complete acquisition of both in-plane locomotion information and out-of-plane posture changes, providing image data for subsequent experimental analysis.

The control unit of the experimental platform mainly consisted of a host computer, an image acquisition and processing program, and a power communication module. It was used to obtain the robot pose, compute the control input, and transmit current commands, thereby providing hardware support for the subsequent gradient-magnetic-field-based force-driven experiments.

In addition, dedicated task environments were constructed to validate the proposed force-driven control method in representative scenarios, including transparent acrylic narrow tunnels and a flexible-membrane interaction platform. The acrylic tunnels were used to simulate confined-space navigation, whereas the flexible membrane setup was used to simulate local occlusion conditions caused by intestinal mucosal folds or similar soft obstructions. Together, these devices constituted the experimental basis for the force-driven motion control study.

### 2.3. Principle of Gradient-Field-Based Actuation

Unlike a uniform magnetic field, which mainly drives robot posture variation through magnetic torque, a gradient magnetic field can exert magnetic force on a magnetic robot and thereby enable force-driven motions such as translation and lifting. By jointly regulating the magnetic flux density and the magnetic field gradient at the robot position, force-driven motion control can be achieved for different task scenarios.

The magnetic force acting on the robot in a non-uniform magnetic field can be expressed as

(1) $$F=\left(m\cdot \nabla \right)B=\left(m_{x}\frac{\partial }{\partial x}+m_{y}\frac{\partial }{\partial y}+m_{z}\frac{\partial }{\partial z}\right)B=\left[\begin{array}{ccc} \frac{\partial B_{x}}{\partial x} & \frac{\partial B_{x}}{\partial y} & \frac{\partial B_{x}}{\partial z}\\ \frac{\partial B_{y}}{\partial x} & \frac{\partial B_{y}}{\partial y} & \frac{\partial B_{y}}{\partial z}\\ \frac{\partial B_{z}}{\partial x} & \frac{\partial B_{z}}{\partial y} & \frac{\partial B_{z}}{\partial z} \end{array}\right]m=Jm,$$

where m is the equivalent magnetic moment of the robot, B is the magnetic flux density at the robot position, and J is the Jacobian matrix of the magnetic field gradient, given by

(2) $$J=\left[\begin{array}{ccc} \frac{\partial B_{x}}{\partial x} & \frac{\partial B_{x}}{\partial y} & \frac{\partial B_{x}}{\partial z}\\ \frac{\partial B_{y}}{\partial x} & \frac{\partial B_{y}}{\partial y} & \frac{\partial B_{y}}{\partial z}\\ \frac{\partial B_{z}}{\partial x} & \frac{\partial B_{z}}{\partial y} & \frac{\partial B_{z}}{\partial z} \end{array}\right].$$

In this study, the midpoint between the two magnetic feet was taken as the robot position. Since the total magnetic field is generated by the superposition of multiple coils, the total magnetic flux density and the total gradient matrix at a working point P can both be obtained by linearly summing the contributions of all coils. For each coil, the magnetic flux density $B\left(P;I\right)$ and the corresponding gradient matrix $J\left(P;I\right)$ at point P were calculated based on the Biot–Savart law, and the total magnetic force acting on the robot at that point was then obtained accordingly.

From the perspective of robot motion control, the magnetic moment direction, the magnetic field direction, and the force direction should be aligned as much as possible. This not only helps concentrate the driving force along the desired motion direction and thus improves motion stability but also enhances the effective force output in the target direction under current-limited conditions. Accordingly, the total magnetic flux density was required to be aligned with the desired direction $\hat{b}$, and the total magnetic force was also constrained to be aligned with $\hat{b}$. This leads to the following linear equation:

(3) $$\left[\begin{array}{ccc} B & -\hat{b} & 0\\ F & 0 & -\hat{b} \end{array}\right]\left[\begin{array}{c} I\\ \lambda_{B}\\ \lambda_{F} \end{array}\right]=0,$$

where I is the current vector of the power supplies, representing the output currents of the corresponding channels, and $\lambda_{B}$ and $\lambda_{F}$ denote the amplitudes of the magnetic flux density and magnetic force, respectively. This equation imposes the collinearity constraints

(4) $$B∥F∥\hat{b},$$

under a given target direction.

Since the coefficient matrix on the left-hand side of the above equation belongs to $R^{6\times 8}$, the system generally has non-unique solutions. Singular value decomposition (SVD) was therefore used to obtain the null-space solution. Let $V_{i}$ denote the null-space basis vectors; the general solution can then be written as

(5) $$\left[\begin{array}{c} I\\ \lambda_{B}\\ \lambda_{F} \end{array}\right]=\underset{i\in L}{\sum }\alpha_{i}V_{i},$$

where L is the index set of the null-space basis vectors and $\alpha_{i}$ are the unknown coefficients.

For a robot at a given state, if a magnetic force with an upper bound of $F_{0}$ is desired along direction $\hat{b}$, the magnetic field at the working point should satisfy

(6) $$\lambda_{B}=\left\|B\right\|\geq B_{0},$$

(7) $$\lambda_{F}=\left\|F\right\|\leq F_{0},$$

where $B_{0}=3\mathrm{mT}$ is introduced to ensure that the robot heading remains aligned with the magnetic field direction, and $F_{0}$ denotes the prescribed upper bound of the magnetic force.

On this basis, the optimal solution satisfying the constraints can be selected from the general solution by linear programming:

(8) $$\underset{\alpha_{i}\in R}{\max}\underset{i\in L}{\sum }\alpha_{i}V_{\left(8|i\right)},$$

subject to

(9) $$-I_{max}\leq \underset{i\in L}{\sum }\alpha_{i}V_{\left(j|i\right)}\leq I_{max},j=1,2,3,4,5,6,$$

(10) $$\underset{i\in L}{\sum }\alpha_{i}V_{\left(7|i\right)}\geq B_{0},$$

(11) $$0\leq \underset{i\in L}{\sum }\alpha_{i}V_{\left(8|i\right)}\leq F_{0},$$

Thus, the current vector satisfying the desired directional requirements can be obtained.

To extract a unique control input from the non-unique general solution, linear programming was further introduced to optimize the solution space under the constraints of current limits, magnetic-field lower bound, and magnetic-force upper bound. In this way, the magnetic field and magnetic force satisfying the desired direction could be determined, and the corresponding current input could be obtained. Because the objective function maximizes $\lambda_{F}$ under a prescribed safe upper bound $F_{0}$, the optimization result is equivalent to approaching $F_{0}$ as closely as possible within the feasible region. During execution, $F_{0}$ can be increased gradually according to the task stage, thereby enabling progressive enhancement of the magnetic force while avoiding abrupt force changes that may induce posture jumps or system instability. Once $F_{0}$ exceeds the system limit, the actual output force can no longer increase because of the current constraint, and further increasing $F_{0}$ will not enhance the generated magnetic force.

With this method, the control current can be solved online according to the robot position and task direction, providing a unified low-level actuation framework for subsequent force-driven actions such as translation, lifting, and posture switching.

### 2.4. Force-Driven Motion Strategies

Based on the above gradient-field actuation principle and control-input solution method, two representative motion strategies, namely translation and lifting, were further developed for different task requirements. Although both strategies rely on the joint regulation of magnetic flux density and magnetic force at the working point, their actuation schemes differ because of the different motion objectives, contact conditions, and environmental forces to be overcome.

#### 2.4.1. Translational Control Strategy

A Pulsed Actuation Method

For navigation tasks in confined spaces, the robot can no longer rely on the conventional periodic crawling gait for propulsion. In narrow spaces, the swinging motion of the magnetic feet and the vertical body undulation are strongly constrained. Therefore, in low-height or confined environments, a translation mode based on gradient-magnetic-force actuation was adopted as the primary propulsion mode.

**Figure 3:**
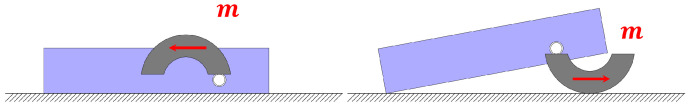
Two robot postures in which the magnetic moment is parallel to the horizontal plane.

To improve motion stability, an appropriate robot posture must first be selected for translational motion. A sliding posture with the magnetic moment direction approximately lying in the horizontal plane was adopted, so that the robot mainly received a driving force along the target direction in the plane under the gradient magnetic field. This posture reduces additional pitch disturbances and avoids increasing the traversal height due to local body lifting, making it more suitable for translation through narrow spaces. As shown in Figure 3, the actual robot structure allows two postures in which the magnetic moment direction remains approximately horizontal. Compared with the posture in which the magnetic moment is aligned with the desired motion direction, the posture with the magnetic moment opposite to the desired motion direction does not significantly increase the overall robot height, and it causes less vibration and wear during sliding. Therefore, the latter was selected as the sliding posture for translational control in the subsequent experiments.

For the actuation scheme, translational motion was realized by pulsed magnetic-force actuation rather than by continuously applying a constant magnetic force. The rationale is that the coefficient of static friction is generally greater than that of kinetic friction between the robot and the substrate, i.e.,

(12) $$F_{s}^{max}=\mu_{s}N>F_{k}=\mu_{k}N,$$

where $\mu_{s}$ and $\mu_{k}$ are the coefficients of static and kinetic friction, respectively. If a constant magnetic force is continuously applied, once the robot starts sliding, the contact resistance changes from static friction to the smaller kinetic friction, which may lead to velocity accumulation, overshoot, and posture deviation, thereby reducing motion controllability. In contrast, pulsed actuation periodically switches between “loading” and “stop” states, so that the robot undergoes only a finite displacement during each actuation cycle and returns to a relatively stable contact state during the off phase. As a result, the overall motion exhibits a quasi-static stepwise translation behavior.

Specifically, during each driving cycle, the control current was first solved online according to the current robot position and the desired motion direction, and a magnetic force along the target direction was generated during the loading phase. Once this force exceeded the static-friction threshold at the contact interface, the robot translated along the target direction. The system then entered the off phase, in which the external driving force was removed and the robot gradually came to rest under friction and damping. By repeating this process, stable translational propulsion could be achieved in confined spaces.

The key control parameters in this translational strategy include the loading time, the off time, and the magnetic-force upper bound $F_{0}$. The loading time determines the displacement generated in each actuation cycle, whereas the off time ensures that the robot sufficiently decelerates and returns to a stable contact state before the next loading phase. The parameter $F_{0}$ determines whether the magnetic force is sufficient to reliably overcome static friction and produce effective motion. By tuning these parameters, a trade-off can be established between propulsion speed and motion stability: a larger $F_{0}$ or a longer loading time tends to improve motion efficiency, but may also increase the risk of side slip, collision with boundaries, or posture disturbance; in contrast, a smaller force level and a shorter step length are more suitable for achieving fine and controllable displacement. In practice, these parameters can be adjusted dynamically to maintain stable robot motion.

B Crawling-to-Translation Mode Switching

The robot requires different actuation modes in different task scenarios. When the spatial condition is relatively open, the robot can achieve stable crawling under a rotating uniform magnetic field through periodic posture variation, where cyclic rotation of the magnetic feet drives the body forward. In contrast, in low-height or narrow spaces, the foot swing and body undulation required by the conventional crawling gait cannot be fully deployed. Under such conditions, a translation mode driven by gradient magnetic force is more suitable. Therefore, to improve the adaptability of the robot to complex environments, mode switching between uniform-field-driven crawling and gradient-field-driven translation is required.

From the viewpoint of actuation mechanism, a rotating uniform magnetic field mainly generates magnetic torque to regulate robot posture and induce cyclic contact motion of the magnetic feet, thereby producing crawling locomotion. In contrast, a gradient magnetic field mainly generates a magnetic force along the desired direction and directly drives translational motion. Accordingly, the switching between these two locomotion modes is essentially a switching between external magnetic-field actuation forms, namely, from torque-dominant rotating uniform magnetic fields to force-dominant gradient magnetic fields.

In practical operation, the robot first approaches the target region in the crawling mode under a rotating uniform magnetic field. When it reaches the entrance of a low-height passage or a location where force-driven manipulation is required, the rotating-field actuation is stopped and the robot posture is adjusted to the sliding posture suitable for translation. Then, the control current corresponding to the gradient magnetic field is solved online according to the robot position and target direction, and the robot is switched to the gradient-force-driven mode to translate along the desired direction. After the confined-space navigation task is completed, a rotating uniform magnetic field can be applied again to restore the posture suitable for crawling, thereby switching the robot back to the crawling mode for subsequent motion. In this way, the robot can switch between crawling and translation on the same experimental platform according to environmental constraints, combining efficient propulsion in open regions with stable navigation in confined spaces.

#### 2.4.2. Lifting Control Strategy

**Figure 4:**
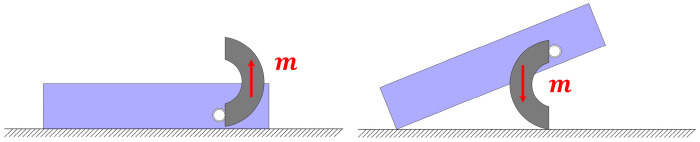
Two robot postures in which the magnetic moment is parallel to the vertical axis.

In addition to translation, a lifting strategy based on gradient magnetic force was further developed to realize the head-up motion of the robot and lifting of a flexible overlying obstacle. Unlike translational motion, which mainly relies on horizontal driving force, the lifting action requires a significant upward force to overcome both the robot’s own weight and the weight of the overlying obstacle. Therefore, both the posture configuration and the actuation scheme for lifting differ from those used in translational motion. To generate an upward magnetic-force component, the magnetic feet are first adjusted to an upright posture. In practice, two upright postures are possible (Figure 4). The posture with the magnetic moment pointing vertically upward is selected because the opposite posture, with the magnetic moment pointing vertically downward, places the robot center of mass at a higher position and is expected to be less stable during lifting. Therefore, the upward-oriented posture was adopted in this study. Under this condition, a vertical magnetic field gradient can produce an upward magnetic force, allowing the front part of the robot body to be gradually lifted while simultaneously elevating the overlying membrane.

Before the lifting action is executed, the robot must first be adjusted to a posture suitable for vertical force generation, such that the equivalent magnetic moment direction of the magnetic feet is aligned as closely as possible with the vertical direction. In this configuration, by controlling the magnetic field direction and the vertical gradient distribution at the working point, an upward magnetic force can be generated to gradually lift the front part of the robot body. Because this action is usually accompanied by continuous variation in the contact state between the robot and the substrate, and may also be affected by the reaction force from the overlying flexible membrane, the lifting process requires smoother force variation than translational motion.

Accordingly, pulsed loading was not adopted for lifting control; instead, a continuous loading strategy was used. Compared with intermittent actuation, continuous loading avoids vibration and posture fluctuation caused by repeated start–stop transitions, allowing the force variation along the vertical direction to remain smoother and thus facilitating stable head-up and lifting motions. In implementation, the control current was still solved online according to the robot position and the desired force direction. However, during execution, the upper bound of the magnetic force $F_{0}$ was increased gradually so that the upward magnetic force acting on the robot rose continuously from a low level. When the upward force was still insufficient to overcome gravity, contact constraints, and the external flexible load, the robot maintained its original posture. As $F_{0}$ increased further, the front part of the robot gradually lifted until the flexible overlying obstacle was raised. At that point, the increase of $F_{0}$ was stopped and the system reached a new stable state.

This progressive loading strategy has two major advantages. First, it avoids abrupt magnetic-force changes that may cause instantaneous jumping, structural impact, or posture instability, thereby improving controllability. Second, it allows the driving force to approach an appropriate level gradually under uncertain external loading conditions, reducing the risk of imposing an excessively large instantaneous force on the overlying flexible object. Correspondingly, after the lifting task is completed, a symmetric progressive unloading process is applied by gradually decreasing $F_{0}$, allowing the robot to return smoothly to its initial state. This unloading strategy prevents sudden rebound or secondary disturbance caused by rapid force removal, thereby improving motion stability and repeatability.

Overall, the translational strategy emphasizes small-step, quasi-static propulsion in confined spaces, whereas the lifting strategy emphasizes smooth loading in the vertical direction and continuous posture variation. Both strategies are established on the same unified framework of gradient magnetic field actuation and control-input solution, but different posture configurations and force-loading schemes are used to achieve force-driven motion control for different task requirements.

## 3. Results and Discussion

### 3.1. Gradient-Field-Driven Navigation in Confined Spaces

To validate the ability of the proposed gradient-magnetic-force-driven method to support navigation in confined spaces, transparent acrylic tunnel experiments were conducted. These experiments were designed to simulate geometrically constrained environments such as low-height passages and to examine whether the robot could stably traverse such environments when the conventional crawling gait could no longer be executed.

**Figure 5:**
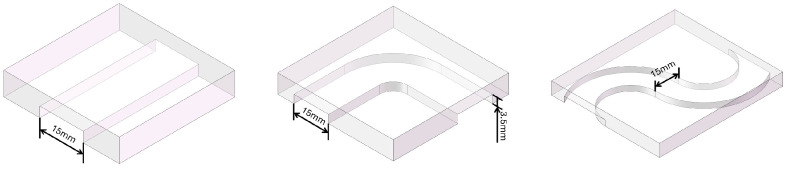
Schematics of the three tunnel geometries: I-shaped tunnel (**left**), L-shaped tunnel (**middle**), and S-shaped tunnel (**right**).

Based on the above strategy, transparent acrylic tunnels were designed with an inner height of 3.5 mm and a width of 15 mm. Since the span of the robot feet is approximately 4 mm, and additional vertical clearance is required for body lifting and foot rolling during crawling, the conventional crawling mode generally requires a passage height of no less than 6 mm for stable execution. Therefore, in the tunnel environment used here, the robot could no longer rely on crawling for propulsion and instead had to switch to the translation mode driven by gradient magnetic force. To cover representative geometric constraints, three tunnel configurations were designed: an I-shaped straight tunnel, an L-shaped right-angle tunnel, and an S-shaped curved tunnel. Their structural schematics and photographs are shown in Figure 5.

The experiments were performed using a control scheme in which the desired force direction was manually specified while the magnetic field and coil currents were solved online by the system. Specifically, the operator manually updated the target direction according to the robot position and tunnel geometry, whereas the system used real-time pose information to calculate the magnetic field and current input satisfying the constraints, thereby producing a gradient magnetic force along the target direction. It should be noted that manual intervention was limited to updating the desired direction and adjusting the duty cycle, whereas the actual calculation of the magnetic field and current input was automatically completed by the previously described control-input solution framework, which reduced the uncertainty associated with manual tuning. In the experiments, the translation mode was driven by pulsed actuation with a duty cycle of 45%. A relatively larger duty cycle was used in straight segments to improve propulsion efficiency, while smaller duty cycles were adopted in turning and curved segments to produce smaller step lengths and improve trajectory controllability. The robot first approached the tunnel entrance in the crawling mode, then adjusted to the sliding posture and switched to gradient-force-driven translation. After passing through the tunnel, it switched back to the crawling mode, recovered its posture, and left the test area.

**Figure 6:**
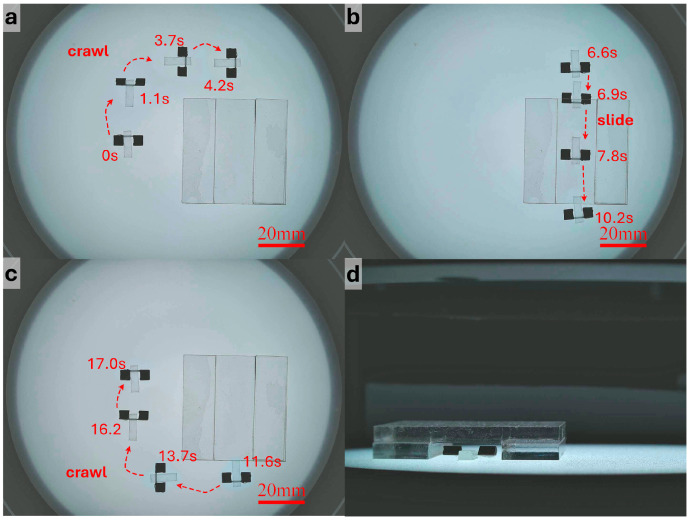
Navigation through the I-shaped tunnel and locomotion mode switching. (**a**) The robot crawls toward the entrance of the I-shaped tunnel. (**b**) The robot passes through the tunnel under gradient-magnetic-force-driven translation. (**c**) After leaving the tunnel, the robot switches back to the crawling mode. (**d**) Side view of the robot passing through the tunnel.

Figure 6 and Video S1 shows the navigation process in the I-shaped straight tunnel. As shown in Figure 6a, the robot used crawling motion to rapidly approach the target region before entering the narrow passage. Because the longitudinal space inside the tunnel was limited, the robot switched to translational motion to pass through the confined section, as shown in Figure 6b, while the non-confined region shown in Figure 6c was still traversed using crawling for more precise control. The side view in Figure 6d further shows that the robot translated through the narrow region while remaining in full contact with the substrate. Since the tunnel direction remained constant and no turning was required in this case, the robot did not need frequent updates of the desired direction after entering the tunnel. Instead, the system continuously solved the magnetic field and coil currents along the tunnel axis according to the real-time pose, enabling stable forward translation. The results indicate that the robot could pass through the I-shaped tunnel at a relatively high speed without obvious collision with the sidewalls, jamming, or posture instability. After exiting the tunnel, the robot switched back to the crawling mode and left the tunnel region, completing the full navigation process.

**Figure 7:**
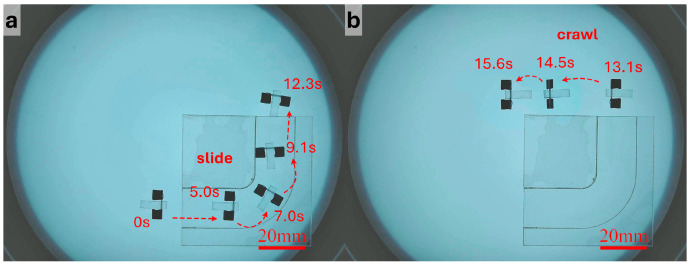
Navigation through the L-shaped tunnel and locomotion mode switching. (**a**) The robot passes through the L-shaped tunnel under gradient-magnetic-force-driven translation. (**b**) After leaving the tunnel, the robot switches back to the crawling mode.

Figure 7 and Video S2 presents the results obtained in the L-shaped right-angle tunnel. The main difficulty in this scenario lies in the need to complete a change in direction within a narrow space while maintaining sufficient forward motion to pass the chamfered corner. Before reaching the turning region, the robot used the same translational parameters as those in the straight segment. Once it approached the corner, the duty cycle was reduced so that each actuation cycle generated a smaller displacement, thereby decreasing the risks of side slip and wall collision. The operator then updated the desired direction more frequently, allowing the robot to complete the turn progressively through alternating posture correction and small forward advances. After entering the second straight segment, a larger $F_{0}$ and duty cycle were restored to improve propulsion efficiency. The experiment shows that the robot could successfully traverse the L-shaped tunnel and switch back to crawling after leaving the exit.

**Figure 8:**
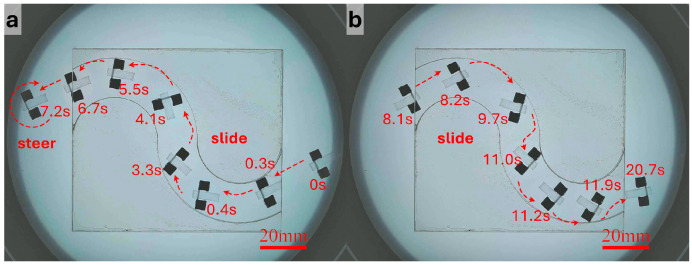
Bidirectional navigation and turning in the S-shaped tunnel. (**a**) After passing through the S-shaped tunnel under gradient-magnetic-force-driven translation, the robot turns around. (**b**) The robot returns along the original path under gradient-magnetic-force-driven translation.

For the S-shaped curved tunnel, the robot needed to continuously adjust its direction under varying curvature, making this scenario the most demanding in terms of control precision. To suppress accumulated error in the curved section and reduce the risk of losing control, more conservative translational parameters were adopted. Specifically, the duty cycle was further reduced compared with that used in the straight segment, so that the robot advanced with smaller step lengths. The operator continuously adjusted the desired direction according to the deviation of the robot from the tunnel centerline, while the system generated the corresponding control currents online. As shown in Figure 8 and Video S3, the robot was able to complete forward traversal of the S-shaped tunnel. To further evaluate repeatability and directional reversibility, the robot rotated approximately 180° at the tunnel exit, re-entered the tunnel, and completed a reverse traversal using the same strategy. These results indicate that, with the combination of small step lengths and frequent directional correction, the robot could maintain controllable motion under curved-path constraints, and it exhibited a certain degree of repeatable bidirectional traversal capability.

Overall, the transparent acrylic tunnel experiments demonstrate that when the tunnel height is insufficient to support the conventional crawling gait, the robot can still achieve stable traversal by switching to the translation mode driven by gradient magnetic force. The I-shaped straight tunnel could be passed rapidly with limited manual intervention, whereas the L-shaped and S-shaped tunnels, which involved changes in direction, required smaller magnetic-force amplitudes or lower duty cycles to produce finer stepwise motion, together with more frequent updates of the desired direction to complete turning and curved navigation. In addition, the transition between crawling and translation was smooth throughout the experiments, with no obvious posture instability or motion interruption, indicating that the proposed actuation-mode-switching strategy is practically feasible. These results verify the effectiveness of the online current-solved gradient-magnetic-force-driven method in geometrically constrained environments and provide the basis for the subsequent flexible-membrane interaction experiments.

### 3.2. Simulated Intestinal Mucosal Elevation

In addition to translational navigation in confined spaces, gradient-magnetic-force-driven actuation can also be used to achieve local interaction between the robot and flexible obstructive objects. In endoscopic scenarios, membrane-like tissues, folds, or attached materials may obstruct the field of view. To evaluate the capability of the robot to create local space expansion through vertical magnetic-force actuation, a simulated intestinal mucosal elevation experiment was designed. This experiment did not represent a complete surgical procedure; rather, it was intended to mimic the typical functional requirement of intestinal mucosal elevation, in which the robot raises an overlying flexible membrane through a head-up motion to create a larger local visible space.

A pair of Maxwell coils was arranged in the vertical direction to generate a relatively large vertical magnetic field gradient in the central region, thereby providing the normal magnetic force required for lifting. Compared with constructing a vertical gradient by differential current control of Helmholtz coils, the Maxwell coils offer higher gradient-generation efficiency and a clearer gradient direction in the vertical axis, making them more suitable for stable vertical lifting control.

**Figure 9:**
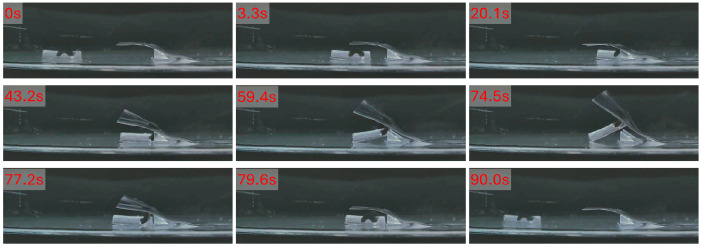
Experiment for simulated intestinal mucosal elevation.

To validate the feasibility of this lifting strategy in a flexible-obstacle interaction task, an experimental setup was constructed, as shown in Figure 9. A PDMS membrane with a thickness of approximately 0.1 mm and a width of 20 mm was used as the flexible obstructive object. To mimic the initially raised state of intestinal mucosa or mucosal folds, two triangular resin supports were placed at the junction between the membrane and the substrate, thereby creating a local occlusion environment with an initial membrane bulge.

The experiment consisted of four stages: approach and positioning, lifting, posture holding, and recovery and departure. First, the robot approached the junction between the membrane and the substrate using the translational strategy driven by horizontal gradient magnetic force, so that it was positioned appropriately for the subsequent head-up motion. During this stage, the desired force direction lay in the horizontal plane, and the control current was solved online according to the real-time robot pose, driving the robot gradually toward the target region. Once the designated position was reached, the robot stopped translating and entered the posture-adjustment stage.

Next, the magnetic flux density direction was gradually adjusted upward so that the magnetic feet progressively stood upright and the robot transitioned to the lifting posture shown in Figure 9 and Video S4. During this process, the membrane exerted a reaction force on the robot, while the friction between the robot and the substrate restricted pure rotational motion. As a result, a slight reverse displacement, i.e., backward motion, was observed. In the experiments, this backward displacement was approximately 4 mm. After the posture transition was completed, the robot entered the lifting stage. At this point, the duty cycle was set to 100%, and the upper bound of the magnetic force $F_{0}$ was continuously increased so that the vertical magnetic force acting on the robot gradually rose. As the driving force increased, the front part of the robot body was lifted slowly, continuously raising the membrane above it. The results show that the membrane could be lifted clearly, and the robot finally reached a stable lifted posture, at which the angle between the robot body and the substrate was approximately 35°.

After the maximum lifting state was reached, $F_{0}$ was kept constant so that the robot maintained the elevated posture for approximately 15 s, in order to evaluate the stability of the action under continuous loading. During this holding phase, the membrane remained clearly elevated and exhibited only limited height fluctuation. According to the variation in the robot pitch angle, the lifting-height fluctuation was estimated to be approximately 5.2 mm, indicating that the robot possessed a certain posture-holding capability during the lifting process.

After the holding phase, $F_{0}$ was gradually reduced using a progressive unloading strategy, allowing the robot to return smoothly to its initial posture while the membrane gradually recovered to its original state. Once the robot had completely returned to the substrate, the magnetic flux density direction was adjusted back to the horizontal direction so that the robot recovered its translational posture. The robot then translated away from the target region under gradient magnetic force, completing the full process of approach, lifting, holding, recovery, and departure. Figure 9 shows the key snapshots of the experiment.

Overall, the simulated intestinal mucosal elevation experiments demonstrate that, under sufficient vertical gradient output, the robot can achieve stable head-up lifting and produce a clear elevating effect on a lightweight flexible membrane when continuous loading together with progressive increase and decrease of $F_{0}$ is used. These results indicate that gradient-magnetic-force-driven actuation can support not only translational navigation in confined spaces but also local interaction with flexible objects, thereby providing a technical basis for functions such as local visual-field expansion in endoscopic assistance scenarios. At the same time, the observed backward displacement suggests that the reaction force from the flexible object still has a pronounced influence on posture adjustment. This issue may be alleviated in future work by introducing a horizontal compensating force component or by employing more refined posture-control strategies to further improve action accuracy and repeatability.

## 4. Conclusions

To address the demand for direct force-driven motion of the robot in confined environments, a gradient-magnetic-field-based force-driven control method was proposed. By establishing the mapping among coil current, magnetic field, and magnetic force at the robot working point, and by solving the control input through singular value decomposition and linear programming, the system could generate effective magnetic force along the desired direction online under current and magnetic-field constraints. This method provided a unified low-level actuation framework for both translational and lifting motions of the robot.

On this basis, two representative force-driven motion strategies were further developed. For planar propulsion in confined spaces, a pulsed magnetic-force actuation strategy was adopted to enable stable quasi-static stepwise translation. For local space expansion, a continuous loading strategy with progressive adjustment of the magnetic-force upper bound was used to realize smooth head-up and lifting motions. These two strategies correspond to different task-dependent force requirements and demonstrate the feasibility of extending robot functionality through gradient-magnetic-force-driven actuation.

Experimental results showed that when the passage height was insufficient for the conventional crawling gait, the robot could stably traverse I-shaped, L-shaped, and S-shaped narrow tunnels by switching to the translation mode driven by gradient magnetic force, demonstrating that the proposed method can effectively support navigation tasks in geometrically constrained environments. Furthermore, in the simulated intestinal mucosal elevation experiments, the robot was able to gradually lift the front part of its body under vertical magnetic-force actuation and produce a clear elevating effect on the overlying PDMS membrane, verifying the feasibility of the proposed method for local interaction with flexible obstructive objects.

Overall, the proposed gradient-magnetic-field-based force-driven control method effectively extends the locomotion capability of the robot, enabling not only its original crawling mode but also direct force-driven translation and vertical lifting. This study demonstrates the potential of magnetic microrobots for navigating low-height confined spaces and interacting locally with flexible objects. The current tunnel and membrane experiments serve as simplified proof-of-concept validations. In future work, the proposed method will be further evaluated in real ex vivo intestinal tissue, which can better reflect the softness, wetness, and deformability of the intestinal environment. Further efforts will also focus on suppressing the backward displacement observed during lifting, improving motion repeatability, and achieving more autonomous control in more complex environments.

## Data Availability

The original contributions presented in this study are included in the article. Further inquiries can be directed to the corresponding author.

## Supplementary Materials

The following supporting information can be downloaded at: https://www.mdpi.com/article/10.3390/mi17040476/s1, Video S1: Navigation through the I-shaped tunnel and locomotion mode switching, Video S2: Navigation through the L-shaped tunnel and locomotion mode switching, Video S3: Bidirectional navigation and turning in the S-shaped tunnel, Video S4: Experiment for simulated intestinal mucosal elevation.

## References

[1] Wang J., Dong R., Wu H., Cai Y., Ren B. (2020). A review on artificial micro/nanomotors for cancer-targeted delivery, diagnosis, and therapy. Nano-Micro Lett..

[2] Luo M., Feng Y., Wang T., Guan J. (2018). Micro-/nanorobots at work in active drug delivery. Adv. Funct. Mater..

[3] Ding J., Venkatesan R., Zhai Z., Muhammad W., Nakkala J.R., Gao C. (2020). Micro-and nanoparticles-based immunoregulation of macrophages for tissue repair and regeneration. Colloids Surf. B Biointerfaces.

[4] Huang C., Xu T., Yu H., Wu X. (2024). A novel H-shaped soft magnetic microrobot for automatic manipulation in dynamic environments. IEEE Trans. Autom. Sci. Eng..

[5] Nan F., Li X., Zhang S., Ng J., Yan Z. (2022). Creating stable trapping force and switchable optical torque with tunable phase of light. Sci. Adv..

[6] Xiao Y., Zhang J., Zhao X., Fang B., Ma L., Hao N. (2023). An artificial acoustics-actuated microrobot bioinspired by Chlamydomonas. Sens. Actuators A Phys..

[7] Ma F., Wang S., Wu D.T., Wu N. (2015). Electric-field–induced assembly and propulsion of chiral colloidal clusters. Proc. Natl. Acad. Sci. USA.

[8] Zhang Y., Hess H. (2021). Chemically-powered swimming and diffusion in the microscopic world. Nat. Rev. Chem..

[9] Díez P., Lucena-Sánchez E., Escudero A., Llopis-Lorente A., Villalonga R., Martinez-Manez R. (2021). Ultrafast directional Janus Pt–mesoporous silica nanomotors for smart drug delivery. ACS Nano.

[10] Li M., Wu J., Lin D., Yang J., Jiao N., Wang Y., Liu L. (2022). A diatom-based biohybrid microrobot with a high drug-loading capacity and pH-sensitive drug release for target therapy. Acta Biomater..

[11] Gao L., Akhtar M.U., Yang F., Ahmad S., He J., Lian Q., Cheng W., Zhang J., Li D. (2021). Recent progress in engineering functional biohybrid robots actuated by living cells. Acta Biomater..

[12] Lee H., Kim D.-i., Kwon S.-h., Park S. (2021). Magnetically actuated drug delivery helical microrobot with magnetic nanoparticle retrieval ability. ACS Appl. Mater. Interfaces.

[13] Nguyen K.T., Go G., Jin Z., Darmawan B.A., Yoo A., Kim S., Nan M., Lee S.B., Kang B., Kim C.S. (2021). A magnetically guided self-rolled microrobot for targeted drug delivery, real-time X-Ray imaging, and microrobot retrieval. Adv. Healthc. Mater..

[14] Huang C., Lai Z., Wu X., Xu T. (2022). Multimodal locomotion and cargo transportation of magnetically actuated quadruped soft microrobots. Cyborg Bionic Syst..

[15] Jiang H., He X., Ma Y., Fu B., Xu X., Subramanian B., Hu C. (2021). Isotropic hedgehog-shaped-TiO_2_/functional-multiwall-carbon-nanotube micromotors with phototactic motility in fuel-free environments. ACS Appl. Mater. Interfaces.

[16] Wang Q., Dong R., Yang Q., Wang J., Xu S., Cai Y. (2020). Highly efficient visible-light-driven oxygen-vacancy-based Cu_2+1_O micromotors with biocompatible fuels. Nanoscale Horiz..

[17] Xu T., Cheng G., Liu C., Li T., Zhang X. (2019). Dynamic assembly of microspheres under an ultrasound field. Chem.–Asian J..

[18] Xu T., Soto F., Gao W., Dong R., Garcia-Gradilla V., Magaña E., Zhang X., Wang J. (2015). Reversible swarming and separation of self-propelled chemically powered nanomotors under acoustic fields. J. Am. Chem. Soc..

[19] Liang X., Mou F., Huang Z., Zhang J., You M., Xu L., Luo M., Guan J. (2020). Hierarchical microswarms with leader–follower-like structures: Electrohydrodynamic self-organization and multimode collective photoresponses. Adv. Funct. Mater..

[20] Wu Y., Fu A., Yossifon G. (2020). Active particles as mobile microelectrodes for selective bacteria electroporation and transport. Sci. Adv..

[21] Yang L., Zhang T., Huang H., Ren H., Shang W., Shen Y. (2023). An on-wall-rotating strategy for effective upstream motion of untethered millirobot: Principle, design, and demonstration. IEEE Trans. Robot..

[22] Zhang Y., Yang H., Yang D., Liu X., Liu Z. (2020). Polynomial profile optimization method of a magnetic petal-shaped capsule robot. Mechatronics.

[23] Rashid M.H.O., Lin F. (2024). Magnetically driven biopsy capsule robot with spring mechanism. Micromachines.

[24] Liu J., Wang Q., Wang H., Li Y., Hu X., Cheng J., Dai Y. (2016). Design and fabrication of a catheter magnetic navigation system for cardiac arrhythmias. IEEE Trans. Appl. Supercond..

[25] Yang L., Sun M., Zhang M., Zhang L. (2023). Multimodal motion control of soft ferrofluid robot with environment and task adaptability. IEEE/ASME Trans. Mechatron..

[26] Sun M., Tian C., Mao L., Meng X., Shen X., Hao B., Wang X., Xie H., Zhang L. (2022). Reconfigurable magnetic slime robot: Deformation, adaptability, and multifunction. Adv. Funct. Mater..

[27] Lan X., Du Y., Liu F., Li G. (2023). Development of microrobot with optical magnetic dual control for regulation of gut microbiota. Micromachines.

[28] Wang W., Yan G., Han D., Meng Y., Pu P. (2020). Design and testing of a novel gastrointestinal microrobot. Biomed. Microdevices.

[29] Le V.H., Rodriguez H.L., Lee C., Go G., Zhen J., Du Nguyen V., Choi H., Ko S.Y., Park J.-O., Park S. (2016). A soft-magnet-based drug-delivery module for active locomotive intestinal capsule endoscopy using an electromagnetic actuation system. Sens. Actuators A Phys..

[30] Kim S.H., Ishiyama K. (2013). Magnetic robot and manipulation for active-locomotion with targeted drug release. IEEE/ASME Trans. Mechatron..

[31] Wang T., Ugurlu H., Yan Y., Li M., Li M., Wild A.-M., Yildiz E., Schneider M., Sheehan D., Hu W. (2022). Adaptive wireless millirobotic locomotion into distal vasculature. Nat. Commun..

[32] Hoang M.C., Le V.H., Nguyen K.T., Nguyen V.D., Kim J., Choi E., Bang S., Kang B., Park J.-O., Kim C.-S. (2020). A robotic biopsy endoscope with magnetic 5-DOF locomotion and a retractable biopsy punch. Micromachines.

[33] Lee W., Nam J., Kim J., Jung E., Kim N., Jang G. (2020). Steering, tunneling, and stent delivery of a multifunctional magnetic catheter robot to treat occlusive vascular disease. IEEE Trans. Ind. Electron..

[34] Wang Q., Du X., Jin D., Zhang L. (2022). Real-time ultrasound doppler tracking and autonomous navigation of a miniature helical robot for accelerating thrombolysis in dynamic blood flow. ACS Nano.

[35] Ye M., Zhou Y., Zhao H., Wang X. (2023). Magnetic microrobots with folate targeting for drug delivery. Cyborg Bionic Syst..

[36] Sun M., Hao B., Yang S., Wang X., Majidi C., Zhang L. (2022). Exploiting ferrofluidic wetting for miniature soft machines. Nat. Commun..

[37] Wang S., Qiu M., Liu J., Yin T., Wu C., Huang C., Han J., Cheng S., Peng Q., Li Y. (2023). Preshaped 4D photocurable ultratough organogel microcoils for personalized endovascular embolization. Adv. Mater..

